# Early Postoperative Analgesic Outcomes Following Pre-Induction Intravenous Ibuprofen in Children Undergoing Hypospadias Repair: A Randomized Controlled Study

**DOI:** 10.3390/children13030342

**Published:** 2026-02-27

**Authors:** Xiaohuan Cui, Jianmin Zhang, Zhengzheng Gao, Jingjing Cai, Fang Wang, Lijing Li, Shanshan Zhang

**Affiliations:** Department of Anesthesiology, Beijing Children’s Hospital, Capital Medical University, National Center for Children’s Health, Beijing 100045, China; cui_xiaohuan@yeah.net (X.C.);

**Keywords:** ibuprofen, hypospadias, pediatric analgesia, perioperative care

## Abstract

**Highlights:**

**What are the main findings?**

- Pre-induction intravenous ibuprofen was associated with a lower proportion of children requiring rescue opioid analgesia in the postanesthesia care unit after hypospadias repair.
- Intravenous ibuprofen was associated with a lower incidence of moderate-to-severe pain in the immediate postoperative period without an increase in adverse events.

**What are the implications of the main findings?**

- Intravenous ibuprofen administered during anesthesia induction may be considered as a potential addition to postoperative pain management strategies for short-duration pediatric urological surgery.
- Integrating pain and emergency delirium scales may improve the interpretation of early postoperative pain outcomes in young children.

**Abstract:**

Background: Hypospadias repair is a pediatric surgical procedure associated with relatively pronounced postoperative pain. However, evidence guiding procedure-specific perioperative analgesic strategies remains limited. Although preoperative intravenous ibuprofen has demonstrated analgesic benefits in other pediatric surgical settings, data specific to pediatric urological surgery are scarce. Methods: In this randomized, double-blind, placebo-controlled trial, 104 children (2–7 years old, American Society of Anesthesiologists [ASA] physical status I–II) scheduled for urethroplasty were randomized to receive either intravenous ibuprofen (10 mg/kg; Group I) or saline (Group C) before anesthesia induction. The primary outcome was the proportion of patients requiring rescue opioid analgesia in the postanesthesia care unit (PACU). Secondary outcomes included postoperative FLACC (Face, Legs, Activity, Cry, Consolability), NRS-11 (the numerical rating scale-11 scale), and PAED (Pediatric Anesthesia Emergence Delirium) scores, repeated rescue analgesia, intraoperative opioid use, the LMA (laryngeal mask airway) removal time, and adverse events. Results: Ninety-three patients completed the study (Group I, *n* = 47; Group C, *n* = 46). The proportion of patients requiring rescue analgesia in the PACU was significantly lower in the ibuprofen group than in the control group (12.77% vs. 30.43%, *p* = 0.038, 95% CI: 0.116, 0.968). Moderate-to-severe pain (FLACC ≥ 4) in the PACU occurred less frequently in the ibuprofen group, whereas incidence of emergence delirium was similar between groups. No significant differences were observed in the pain scores on postoperative days 1 and 2, intraoperative opioid use, the LMA removal time, or adverse events. Conclusions: Pre-induction intravenous ibuprofen reduced early postoperative rescue analgesia requirements without increasing adverse events in children undergoing hypospadias repair.

## 1. Introduction

Hypospadias repair and pediatric urethroplasty often result in significant postoperative pain [1]. However, perioperative analgesia remains frequently inadequate [2]. Insufficient postoperative pain management can lead to increased opioid consumption, prolonged recovery, and a higher incidence of complications [3].

Available analgesic options include systemic medication, caudal blocks, and peripheral nerve blocks. Although systemic opioids are effective, they are associated with adverse effects such as nausea, vomiting, constipation, and respiratory depression [4]. Caudal blocks are commonly used but carry risks including block failure, blood aspiration, dural puncture, cardiac arrest, seizures, and sacral pain [5,6]. Pudendal nerve blocks provide localized analgesia with reduced opioid requirements. However, their use is limited by anatomical variability, requiring advanced skills and imaging for proper placement [7].

Preoperative intravenous administration of nonsteroidal anti-inflammatory drugs (NSAIDs) has been shown to reduce postoperative pain and limit opioid use and related side effects in adults [8,9]. However, only a limited number of intravenous NSAID formulations have been approved for pediatric use [10]. Several studies have demonstrated that preoperative intravenous ibuprofen effectively reduces postoperative pain scores in children undergoing tonsillectomy, underscoring its potential for managing acute postoperative pain [11,12]. Nevertheless, differences in surgical characteristics and postoperative pain profiles may limit the direct extrapolation of these findings to other pediatric procedures, particularly urological surgeries involving urethral reconstruction. This study aimed to evaluate whether preoperative intravenous ibuprofen can effectively reduce postoperative pain and opioid consumption in children undergoing hypospadias repair.

## 2. Materials and Methods

### 2.1. Trial Design

This study was designed as a prospective, randomized, double-blind, placebo-controlled, single-center clinical trial with two parallel groups allocated in a 1:1 ratio to receive either intravenous ibuprofen or placebo before anesthesia induction. The trial was registered in the Chinese Clinical Trial Registry (ChiCTR2500101660) after participant enrollment.

### 2.2. Ethics Approval and Study Conduct

This study was conducted exclusively at Beijing Children’s Hospital, Capital Medical University and was approved by the Ethics Committee (approval no. [2023]-E-111-Y; 30 August 2023). Written informed consent was obtained from the parents or legal guardians of all participants prior to enrollment. The trial was conducted in accordance with the Declaration of Helsinki and reported in compliance with the CONSORT guidelines.

### 2.3. Participants and Eligibility Criteria

#### 2.3.1. Inclusion Criteria

Children aged 2–7 years with ASA I–II and scheduled for elective urethroplasty were eligible for inclusion.

#### 2.3.2. Exclusion Criteria

The exclusion criteria included emergency surgery, an expected operative duration longer than 2 h due to the 1.5–2 h half-life of intravenous ibuprofen, an anticipated postoperative hospitalization shorter than 48 h, use of analgesics or sedatives within 24 h before surgery, known allergy or hypersensitivity to ibuprofen or other NSAIDs, and a history of gastrointestinal disease or active gastrointestinal bleeding.

### 2.4. Handling of Participants and Impact on Study Power

We carefully considered the potential impact of excluding patients on study bias and power. To address this, we included all patients who completed the primary outcome, regardless of whether they were lost to follow-up or withdrew from the study, in the intention-to-treat (ITT) analysis. This approach was intended to minimize bias and preserve the integrity of randomization. Additionally, it aimed to ensure that the study power would not be unduly influenced by these withdrawals or exclusions. By including all patients with primary outcome, we aimed to enhance the reliability of our results.

### 2.5. Patient and Public Involvement

The patients and/or the public were not involved in the design, conduct, reporting, or dissemination of the study. The primary and secondary outcomes were selected entirely by the investigators based on clinical relevance.

### 2.6. Standardized Procedure

#### 2.6.1. Induction and Maintenance of Anesthesia

An intravenous line was established in the preoperative waiting area and no premedication was administered. Standard anesthesia monitoring, including continuous electrocardiography, blood oxygen saturation (SpO_2_), bispectral index (BIS), and non-invasive blood pressure, was initiated before induction, with values recorded every 5 min. In the operating room, patients in the ibuprofen group (Group I) received 10 mg·kg^−1^ of intravenous ibuprofen (maximum 400 mg; Easton Biopharmaceuticals, China), diluted to 50 mL for children ≤ 20 kg or 100 mL for those >20 kg, infused over at least 10 min prior to anesthesia induction. The control group (Group C) received an equal volume of normal saline.

All patients underwent standardized anesthesia induction with propofol (2 mg·kg^−1^), cisatracurium (0.1 mg·kg^−1^), and fentanyl (2 mcg·kg^−1^), followed by LMA (laryngeal mask airway) insertion. Anesthesia was maintained with continuous intravenous infusion of propofol (6–10 mg·kg^−1^·h^−1^) and remifentanil (0.2–0.4 mcg·kg^−1^·min^−1^). The bispectral index (BIS) was maintained between 40 and 60, and the hemodynamic parameters were controlled within ±20% of baseline.

At the end of the surgery, the anesthetic agents were discontinued. A continuous infusion analgesia pump containing fentanyl (20 mcg·kg^−1^), tropisetron (0.2 mg·kg^−1^), and normal saline to a total volume of 100 mL was infused at a rate of 2 mL·h^−1^. The LMA was removed once spontaneous breathing resumed and SpO_2_ remained ≥95% for at least 5 min. The patients were then transferred to the postanesthesia care unit (PACU).

#### 2.6.2. Postoperative and Rescue Analgesia

**Figure 1:**
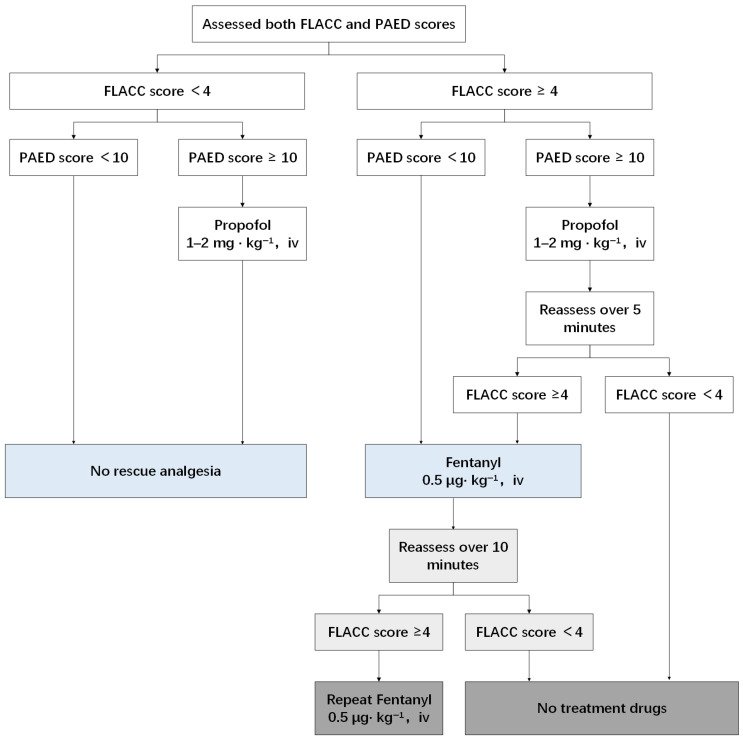
Treatment procedure in the PACU.

In the PACU, postoperative pain and emergence delirium were assessed using the FLACC and PAED scales by a trained, blinded study member at predefined time points. The rescue analgesia strategy followed a standardized stepwise protocol based on the combined FLACC and PAED scores (Figure 1).

Children with FLACC scores of 1–3 and PAED scores of <10 were considered comfortable and required no intervention. Those with FLACC scores of 1–3 but PAED scores ≥ 10 were treated for emergence delirium with propofol (1–2 mg·kg^−1^).

For children presenting with FLACC scores ≥ 4 and PAED scores < 10, fentanyl (0.5 mcg·kg^−1^) was administered for analgesia. When both FLACC scores ≥ 4 and PAED scores ≥ 10 were observed, propofol (1–2 mg·kg^−1^) was administered first. FLACC scores were reassessed 5 min later, and fentanyl (0.5 mcg·kg^−1^) was given if the reassessed FLACC score remained ≥4, regardless of the PAED score.

Repeat fentanyl doses were permitted at intervals of ≥10 min if pain persisted.

In the general ward, pain management during the first 48 h postoperatively begins with identifying the underlying cause, such as surgical site pain, referred pain from postoperative diarrhea or constipation, and irritation caused by the urinary catheter. Based on the identified cause, treatment was provided, including analgesics (ibuprofen/tramadol), anti-infective agents, antidiarrheal or laxative treatments, and, when necessary, fluid management and antispasmodic medications.

### 2.7. Study Outcomes

The primary outcome was the proportion of patients who required postoperative fentanyl rescue in the PACU. Figure 1 illustrates the rescue analgesia procedure. If the FLACC score was 4 or higher and the PAED score was less than 10, the child received 0.5 mcg·kg^−1^ fentanyl. If the FLACC score was 4 or greater and the PAED score was 10 or higher, the child first received 1–2 mg·kg^−1^ propofol and was reassessed 5 min later. If the reassessed FLACC score remained between 4 and 10, 0.5 mcg·kg^−1^ fentanyl was administered, regardless of the PAED score.

The secondary outcomes were as follows:

1. The proportion of patients with a postoperative FLACC score of 4 or greater.
2. The proportion of patients with a postoperative PAED score of 10 or higher.
3. The proportion of patients requiring repeat fentanyl in the PACU.
4. Pain scores on postoperative days 1 and 2 were assessed using two validated scales: the FLACC scale for all patients and NRS-11 reported by caregivers.
5. Intraoperative remifentanil consumption.
6. The LMA removal time, defined as the interval from the discontinuation of maintenance anesthetic agents to removal of the laryngeal mask airway.
7. The incidence of adverse events, including nausea, vomiting, constipation, intra- and postoperative bleeding, and abdominal bloating, was also recorded.

### 2.8. Sample Size

A pilot study reported 64% and 36% rescue analgesia rates in the control and ibuprofen groups, respectively. To detect this difference with α = 0.05 and a power of 0.80 (two-sided), a sample size of 94 patients was needed. Considering a 10% attrition rate, 104 patients were enrolled in this study. Sample size was calculated using PASS software (version 15.0; NCSS, LLC, Kaysville, UT, USA).

### 2.9. Randomization and Blinding

The participants were randomized in a 1:1 ratio using computer-generated block randomization with a block size of four implemented in Microsoft Excel. The allocation was concealed in sequentially numbered, opaque, sealed envelopes. Medications were prepared by an independent pharmacist, and both the ibuprofen solution and saline placebo were identical in appearance. The anesthesiologists, surgeons, caregivers, outcome assessors, and patients were blinded to the group assignment throughout the study.

### 2.10. Statistical Analysis

No imputation of missing data was performed since the number of missing cases was small and occurred randomly. Therefore, analyses were conducted using only complete case data.

Data were analyzed using SPSS version 25.0 (IBM Corp., Armonk, NY, USA). Normality was assessed using the Shapiro–Wilk test. Continuous variables are expressed as the mean ± standard deviation or median (Q1, Q3), and comparisons were made via Student’s t-test or the Mann–Whitney U test, as appropriate. Categorical variables were presented as counts and percentages, and comparisons were made using the chi-square test or Fisher’s exact test. All analyses were two-sided, and *p* < 0.05 was considered statistically significant.

## 3. Results

Participant recruitment was conducted between October 2023–November 2024. All participants were followed up for the assessment of early postoperative outcomes during the immediate postoperative period and on postoperative days 1 and 2.

**Figure 2:**
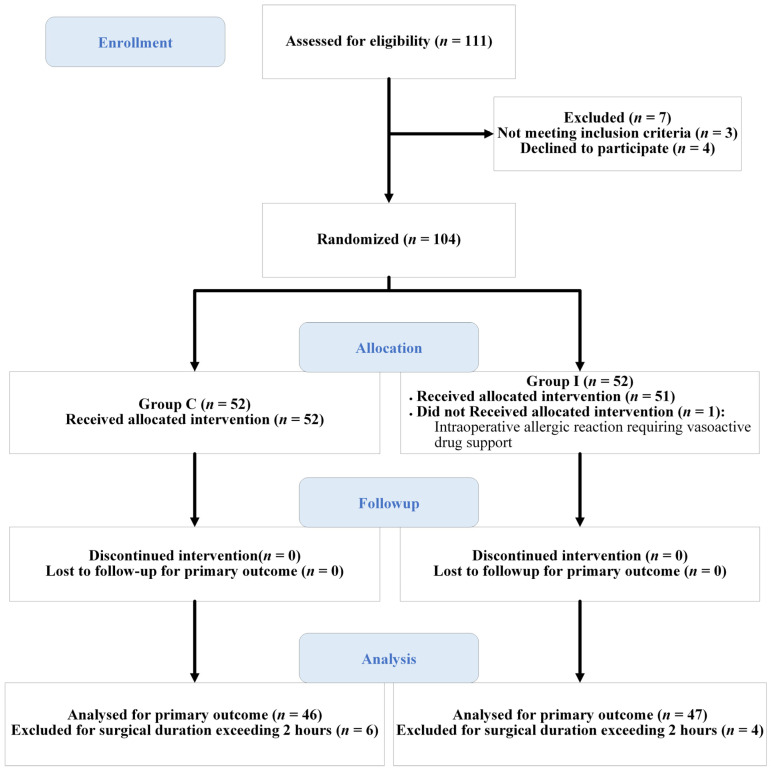
CONSORT flow diagram.

A total of 104 children were randomly assigned to either Group I (*n* = 52) or Group C (*n* = 52). One patient in Group I did not receive the allocated intervention due to an intraoperative allergic reaction requiring vasoactive drug support, and the surgery was canceled. Ten patients (four in Group I and six in Group C) were excluded due to a prolonged surgical duration exceeding 2 h and lack of follow-up on postoperative days 1 and 2. Ultimately, 103 patients (51 in Group I and 52 in Group C) were included in the intention-to-treat (ITT) analysis, while 93 patients (47 in Group I and 46 in Group C) were included in the per-protocol (PP) analysis. The detailed participant flow is presented in the CONSORT flow diagram (Figure 2). Baseline demographic characteristics (age, weight, height, and body mass index) were comparable between the two groups. Other baseline clinical characteristics were analyzed in the per-protocol population and no significant differences were observed between the two groups (Table 1).

### 3.1. Efficacy Outcomes Based on Per-Protocol Analysis

#### 3.1.1. Rescue Analgesia

In Group I, five patients initially presented with a FLACC score of 4 or higher and a PAED score below 10. All children achieved FLACC scores of <4 following fentanyl administration. Another two patients presented with a FLACC score of 4 or higher and a PAED score of 10 or higher. After propofol sedation, one of these patients continued to have a FLACC score between 4 and 10; however, the score decreased to below 4 after fentanyl administration.

In Group C, 13 patients initially presented with a FLACC score of 4 or higher and a PAED score below 10. All patients achieved FLACC scores less than 4 after fentanyl administration. Another four patients had FLACC scores between 4 and 10 with a PAED score of 10 or higher. After propofol sedation, one patient continued to have an FLACC score of 4 or greater and required fentanyl, after which the score decreased to below 4.

The overall incidence of rescue analgesia was 12.77% (6/47) in Group I and 30.43% (14/46) in Group C (*p* = 0.038, 95% CI: 0.116, 0.968) (Table 2).

**Figure 3:**
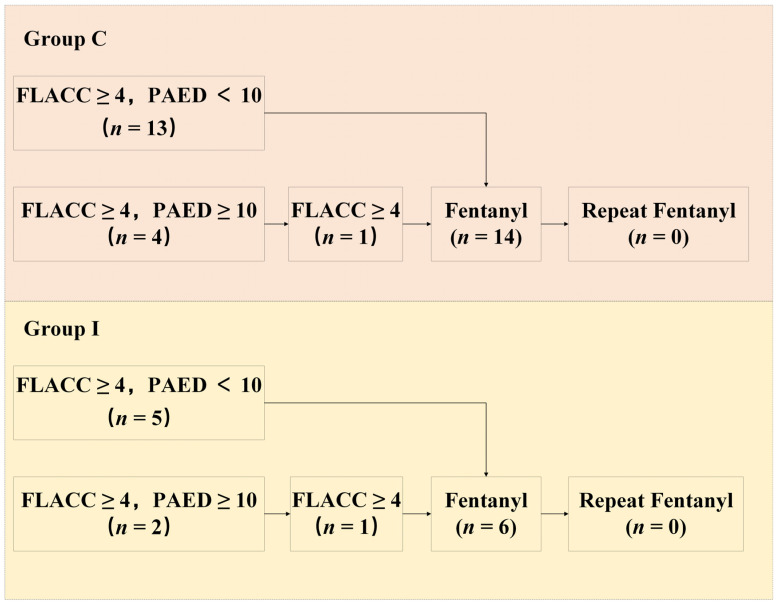
Assessment process and detailed outcomes of rescue analgesia.

None of the patients in either group required more than one rescue intervention. The assessment process and detailed outcomes of rescue analgesia are shown in Figure 3.

#### 3.1.2. Postoperative Pain and Emergence Behavior

In the PACU, the incidence of a PAED score of 10 or higher was comparable between the two groups (Group I: 5/47 [10.64%] vs. Group C: 5/46 [10.87%]; *p* = 1, 95% CI: 0.263, 3.626). However, the incidence of a FLACC score of 4 or higher was significantly lower in Group I than in Group C (7/47 [14.89%] vs. 17/46 [36.96%]; *p* = 0.015, 95% CI: 0.110, 0.813).

On postoperative day 1, patients in Group I had lower rates of FLACC scores between 4 and 10 (1/47 [2.13%] vs. 5/46 [10.87%]; *p* = 0.11, 95% CI: 0.020, 1.589) and NRS-11 scores of 4 or higher (2/47 [4.26%] vs. 6/46 [13.04%]; *p* = 0.16, 95% CI: 0.057, 1.552), although these differences did not reach statistical significance. On postoperative day 2, a FLACC score between 4 and 10 was observed in four of 47 patients (8.50%) in Group I and two of 46 patients (4.30%) in Group C (*p* = 0.68, 95% CI: 0.356, 11.761). Similarly, NRS-11 scores of 4 or higher were reported in four of the 47 patients (8.50%) in Group I and in two of the 46 patients (4.30%) in Group C (*p* = 0.68, 95% CI: 0.356, 11.761).

#### 3.1.3. Intraoperative Medication and Recovery

There were no significant differences between the groups in terms of intraoperative remifentanil consumption (*p* = 0.71) or the LMA removal time (*p* = 0.37).

### 3.2. Safety Outcomes

There was no difference in surgical blood loss between the two groups (*p* = 0.71). Postoperative nausea occurred in one patient in Group C. The patient developed nausea on postoperative day 2, accompanied by low-grade fever, which was attributed to a suspected gastrointestinal infection. The patient’s condition improved after symptomatic treatment, and no other adverse events were reported.

## 4. Discussion

The preoperative administration of intravenous ibuprofen has been shown to attenuate early postoperative pain across a range of surgical settings [11,12,13]. In line with these findings, the present trial demonstrated that pre-induction intravenous ibuprofen reduced the need for rescue analgesia in the immediate postoperative period among children undergoing hypospadias repair. Rather than focusing on the introduction of a new analgesic agent, this study provides procedure-specific clinical evidence supporting the integration of an established non-opioid analgesic into a standardized perioperative analgesic strategy for pediatric urological surgery. From a pharmacological perspective, ibuprofen is more effective at preventing nociceptive sensitization than treating established pain [14]. This provides a rationale for its administration before surgical stimulation and supports its use in this specific pediatric urological procedure.

Although intravenous ibuprofen has been evaluated in various pediatric surgical settings, relatively few studies have focused on pediatric urological procedures. The analgesic effects of preoperative intravenous ibuprofen have been demonstrated in pediatric otolaryngological procedures [11,12]. However, its applicability in pediatric urological surgery is not well established. Therefore, differences in surgical characteristics and postoperative pain profiles, including tissue type and nociceptive mechanisms, may limit the direct extrapolation of findings across procedures. Against this background, the present study added procedure-specific data derived from a standardized anesthetic protocol. It also incorporated age-appropriate assessments of pain and emergence delirium to support the clinical interpretability of early postoperative behavioral responses in this population.

The rapid onset and elimination half-life of intravenous ibuprofen (approximately 2 h) make it well-suited for short-duration procedures. As a result, administration during anesthesia induction may provide effective analgesic coverage during the critical immediate recovery period, though it may not affect later pain outcomes. In this study, even after including patients with surgery durations exceeding 2 h in the ITT analysis, ibuprofen still showed a significant analgesic effect. This suggests that its use in longer procedures may offer some potential in alleviating early postoperative pain.

Postoperative pain management in children is a complex issue, closely related to the type of surgery and postoperative care, especially during the extended wound healing period. The choice of analgesics and routes of administration depends on the severity of pain and the underlying cause. Acetaminophen, NSAIDs, weak opioids like tramadol, and opioids have been widely used. This study did not provide detailed statistics or classification on pain assessment and total analgesic use during the first 24 and 48 h postoperatively. This is because, in hypospadias patients, multiple factors can contribute to pain during the recovery period, not all of which require additional analgesics. For example, some children had a FLACC score of <3 during the first 24 h but showed increased distress at 48 h. Upon assessing their urine output and color, it was found to be related to inadequate fluid intake, leading to urinary catheter irritation. After hydration and antispasmodic treatment, the symptoms were alleviated. These complexities introduced potential bias, which is why cumulative opioid consumption in the first 24 and 48 h was not measured. Non-opioid and weak opioid analgesics are preferred in the ward due to their advantages in avoiding respiratory depression and addiction risks. However, the optimal drug choice, formulation, and timing remain debated [15]. A meta-analysis in adults showed that preemptive acetaminophen reduced opioid consumption within the first 24 h after surgery [16]. However, results in children are more controversial [17,18]. Ibuprofen is well tolerated in infants and children when dosed based on weight. Oral formulations are convenient, while intravenous formulations act faster. Rectal administration, however, may be affected by factors such as suppository expulsion, leading to variable absorption [10,19].

In the early recovery phase, young children often exhibit abnormal behaviors such as crying and agitation, which can lead to clinical confusion. Emergence delirium (ED), agitation, and postoperative pain share overlapping behavioral signs, making it difficult to differentiate between them [20]. A study found that a score of ≥10 on the PAED scale is the best discriminator between the presence and absence of clinical agitation [21]. And evidence suggests that ED can occur in pain-free children [22]. Therefore, we used the PAED scale to assess whether the children exhibited ED, helping to distinguish their neurobehavioral symptoms from early postoperative pain [23]. The FLACC scale [24] shares some overlap with the PAED scale, particularly in the “consolability” domain, whereas behaviors such as “crying” or “uneasy legs” in the FLACC scale were categorized under “restlessness” in the PAED scale [25]. Therefore, when a FLACC score of ≥4 was observed, analgesics were not administered immediately. Instead, in children presenting with both a FLACC score ≥ 4 and a PAED score ≥ 10, propofol was administered first, followed by reassessment of the FLACC score after the child had regained consciousness. This approach was adopted to minimize misclassification rather than to evaluate delirium as the primary outcome. Using this strategy, the incidence of emergence delirium was similar between the two groups. It was relatively low compared with that reported in previous studies on emergence delirium [26,27,28]. This may be attributed to factors such as patient age, type of surgery, and anesthesia maintenance approach.

This study used total intravenous anesthesia, which reduced the incidence of emergent delirium [28], thereby minimizing its impact on early postoperative pain assessment. However, no significant difference in intraoperative remifentanil consumption was observed between the two groups. This finding may be explained by the multifactorial nature of intraoperative hemodynamic fluctuations and the complexity of anesthetic management. Although specific sedation and analgesia targets have been established, intraoperative variations in blood pressure and heart rate are influenced by multiple factors. These include perioperative fluid management, surgical stimulation, and titration with propofol and remifentanil [29]. Moreover, anesthesiologists’ real-time assessment and response to these changes inevitably introduce variability, indicating that remifentanil consumption may not reliably reflect intraoperative nociceptive levels. Additionally, propofol itself exhibits intrinsic analgesic properties [30,31], which may further confound the relationship between remifentanil dose and intraoperative pain intensity. To effectively compare the impact of ibuprofen on intraoperative opioid consumption, a more rigorously designed and easily implementable anesthesia maintenance protocol is essential.

The pain scores on postoperative days 1 and 2 were similar between the two groups, consistent with previous studies [32]. Despite the use of postoperative analgesia pumps, a subset of patients still experienced pain scores of 4 or higher, suggesting that regular postoperative NSAID use may warrant further consideration. On postoperative day 2, a numerically higher proportion of patients in the experimental group exhibited pain scores ≥ 4, compared to the control group. However, this difference did not reach statistical significance. In younger children, postoperative discomfort during the later stages of recovery may reflect a combination of factors beyond surgical pain. These include urinary catheter–related irritation, unfamiliar hospital environments, protective immobilization, restricted activity, and limited opportunities for play. These factors may influence longer-term pain scores. In this study, postoperative pain was assessed using both the FLACC scale, as reported by healthcare providers, and the NRS-11 scale, as reported by caregivers. This dual assessment approach was chosen because healthcare providers can assess pain only at a specific moment, whereas caregivers can estimate pain based on a child’s behavior throughout the day. Pain-monitoring apps guiding caregivers to use the NRS-11 scale for children aged 0–8 years were shown to be feasible [33]. We found that caregiver-reported pain scores were generally higher than those reported by healthcare providers, with a typical difference of no more than two points. Caregivers reported that children were more distressed at night, possibly because daytime distractions, including play, cartoons, or preferred foods, helped alleviate their perceived pain. These findings suggest that assessing postoperative pain in younger children requires a more holistic approach. This approach should integrate factors such as nutrition, mobility, and overall comfort to improve recovery during the recovery period following surgery.

The safety profile of ibuprofen in our study aligns with previous research. The most common adverse effects of NSAIDs are gastrointestinal, including nausea, vomiting, constipation, and bloating. In our study, each patient received a continuous infusion analgesia pump with tropisetron to manage nausea. Postoperative monitoring is essential, and gastroprotective treatments like antacids, H2 blockers, or proton pump inhibitors (PPIs) may be considered based on symptoms. Unlike non-selective NSAIDs, ibuprofen does not significantly impair platelet function. A Cochrane review of 13 randomized controlled trials involving nearly 1000 children found no significant difference in postoperative bleeding between NSAIDs and placebo or other analgesics [34]. Studies on tonsillectomy, skin grafts, and burn injuries also support the safety of ibuprofen without increased bleeding risk [35,36,37]. While bleeding risk is low, interventions should be available. For mild wound oozing, local pressure or hemostatic agents may be applied as needed, while severe bleeding may require reoperation. For gastrointestinal bleeding risks, early identification of risk factors, such as comorbidities, corticosteroid use, and H. pylori infection, is essential [19].

This study had several limitations that should be interpreted with caution. First, the relatively small sample size may limit the generalizability of our findings. Additionally, this trial focused exclusively on a single surgical procedure (hypospadias repair), which may not fully represent the broader pediatric population or other types of pediatric surgeries. Although the study was adequately powered to detect differences in the primary outcome of rescue analgesia, a larger and more diverse cohort is needed to draw more robust conclusions. This is particularly true for secondary outcomes, such as pain scores on postoperative days 1 and 2. Second, although both the FLACC and NRS-11 were used to assess postoperative pain, variability in pain reporting by caregivers and healthcare providers may have introduced some measurement variability. Third, pain assessment in the PACU was performed by a single-blinded observer; while blinding reduces the risk of bias, the absence of multiple independent assessors may have influenced the reliability of the assessments. Fourth, the secondary outcomes (pain scores on postoperative days 1 and 2) were reported without adjustment for multiple comparisons. This increases the risk of Type I error, which may affect the reliability of the findings. Furthermore, although no significant differences in intraoperative remifentanil consumption were observed between the groups. This outcome may have been influenced by uncontrolled perioperative factors, including differences in fluid management, surgical stimulation, and interactions among anesthetic agents. The absence of a standardized hydration protocol in our study is another limitation. Adequate hydration has been shown to impact both postoperative pain and emergence delirium (ED). Future studies should incorporate hydration protocols to better understand their role in perioperative care. Finally, this study did not evaluate long-term outcomes, such as persistent postoperative pain or functional recovery. These outcomes are increasingly recognized as important endpoints in pediatric anesthesia research.

Future studies are needed with larger, multicenter cohorts and multiple independent outcome assessors. Extended follow-up periods are also necessary to validate the findings presented in this study and better define the role of intravenous ibuprofen in multimodal analgesia strategies for pediatric surgery.

## 5. Conclusions

In conclusion, the results of this study suggest that preoperative intravenous ibuprofen may offer benefits in pediatric patients undergoing hypospadias repair. It reduced the need for rescue analgesia in the PACU without increasing drug-induced adverse events. Therefore, it may be considered as a potential addition to postoperative pain management strategies. However, further studies are needed to confirm these findings.

## Figures and Tables

**Table 1:** Baseline demographic and clinical characteristics.

	Group C	Group I	*p*
Age, year *	3.0 (2.0, 4.3)	3.0 (2.0, 4.0)	0.34
Weight, kg *	15.3 (12.8, 17.6)	14.3 (13.0, 17.7)	0.84
High, cm *	95.5 (88.8, 102.3)	94.0 (90.0, 102.0)	0.98
BMI	17.03 ± 2.18	17.01 ± 2.74	0.98
Surgery duration, min *	65.0 (45.0, 94.3)	70.0 (55.0, 91.0)	0.70
The LMA removal time, min **	18.0 (15.0, 23.5)	19.0 (16.0, 25.0)	0.37
Blood loss, mL *	5.0 (2.8, 5.0)	5.0 (3.0, 5.0)	0.71
Mean remifentanil, μg·kg^−1^·min^−1^	0.26 ± 0.03	0.27 ± 0.03	0.71

Abbreviations: Group C, control group; Group I, ibuprofen group. * Some continuous variables are presented as the median (Q1, Q3) because they were not normally distributed, as determined by the Shapiro–Wilk test. ** The LMA removal time, min: the time from discontinuation of maintenance anesthetic agents to removal of the laryngeal mask airway.

**Table 2:** Postoperative pain assessments.

	Per-Protocol (PP) Analysis		Intent-To-Treat Analysis	
	Group C (*n* = 46)	Group I (*n* = 47)	Group C (*n* = 52)	Group I (*n* = 51)
Incidence of rescue fentanyl, *n* (%)				
	14 (30.43%)	6 (12.77%)	17(32.69%)	7(13.73%)
*p*-value	0.038		0.023	
Odds Ratio (OR)	0.334		0.328	
95% Confidence Interval	(0.116, 0.968)		(0.122, 0.878)	
Risk Difference (RD)	−17.66%		−18.96%	
Incidence of a FLACC score ≥ 4, *n* (%)				
	17 (36.96%)	7 (14.89%)	21 (40.38%)	9 (17.65%)
*p*-value	0.015		0.011	
Odds Ratio (OR)	0.299		0.316	
95% Confidence Interval	(0.110, 0.813)		(0.128, 0.785)	
Risk Difference (RD)	−22.07%		−22.73%	
Incidence of a PAED score ≥ 10, *n* (%)				
	5 (10.87%)	5 (10.64%)	7 (13.46%)	6 (11.76%)
*p*-value	1		0.795	
Odds Ratio (OR)	0.976		0.857	
95% Confidence Interval	(0.263, 3.626)		(0.267, 2.751)	
Risk Difference (RD)	−0.23%		−1.70%	
Incidence of a FLACC score ≥ 4 on day 1, *n* (%)				
	5 (10.87%)	1 (2.13%)	*	
*p*-value	0.11			
Odds Ratio (OR)	0.178			
95% Confidence Interval	(0.020, 1.589)			
Risk Difference (RD)	−8.74%			
Incidence of a NRS-11 score ≥ 4 on day 1, *n* (%)				
	6 (13.04%)	2 (4.26%)	*	
*p*-value	0.16			
Odds Ratio (OR)	0.296			
95% Confidence Interval	(0.057, 1.552)			
Risk Difference (RD)	−8.78%			
Incidence of a FLACC score ≥ 4 on day 2, *n* (%)				
	2 (4.30%)	4 (8.50%)	*	
*p*-value	0.68			
Odds Ratio (OR)	2.047			
95% Confidence Interval	(0.356, 11.761)			
Risk Difference (RD)	4.20%			
Incidence of a NRS-11 score ≥ 4 on day 2, *n* (%)				
	2 (4.30%)	4 (8.50%)	*	
*p*-value	0.68			
Odds Ratio (OR)	2.047			
95% Confidence Interval	(0.356, 11.761)			
Risk Difference (RD)	4.20%			

Abbreviations: Group C, control group; Group I, ibuprofen group. * Ten patients with surgery durations exceeding 2 h were included in the ITT analysis, although they missed follow-up on days 1 and 2.

## Data Availability

The data supporting the findings of this study are available from the corresponding author upon reasonable request owing to ethical and privacy restrictions involving pediatric participants.

## Abbreviations

The following abbreviations are used in this manuscript:

ASA	American Society of Anesthesiologists
PACU	postanesthesia care unit
FLACC	Face, Legs, Activity, Cry, Consolability
NRS-11	numerical rating scale-11
PAED	Pediatric Anesthesia Emergence Delirium
LMA	laryngeal mask airway
NSAID	nonsteroidal anti-inflammatory drug
BIS	bispectral index
SpO_2_	blood oxygen saturation
